# Occurrence of Medication Errors and Comparison of Manual and Computerized Prescription Systems in Public Sector Hospitals in Lahore, Pakistan

**DOI:** 10.1371/journal.pone.0106080

**Published:** 2014-08-28

**Authors:** Muhammad Kashif Riaz, Furqan Khurshid Hashmi, Nadeem Irfan Bukhari, Mohammad Riaz, Khalid Hussain

**Affiliations:** University College of Pharmacy, University of the Punjab, Lahore, Pakistan; Federico II University of Naples, Italy

## Abstract

The knowledge of medication errors is an essential prerequisite for better healthcare delivery. The present study investigated prescribing errors in prescriptions from outpatient departments (OPDs) and emergency wards of two public sector hospitals in Lahore, Pakistan. A manual prescription system was followed in Hospital A. Hospital B was running a semi-computerised prescription system in the OPD and a fully computerised prescription system in the emergency ward. A total of 510 prescriptions from both departments of these two hospitals were evaluated for patient characteristics, demographics and medication errors. The data was analysed using a chi square test for comparison of errors between both the hospitals. The medical departments in OPDs of both hospitals were the highest prescribers at 45%–60%. The age group receiving the most treatment in emergency wards of both the hospitals was 21–30 years (21%–24%). A trend of omitting patient addresses and diagnoses was observed in almost all prescriptions from both of the hospitals. Nevertheless, patient information such as name, age, gender and legibility of the prescriber’s signature were found in almost 100% of the electronic-prescriptions. In addition, no prescribing error was found pertaining to drug concentrations, quantity and rate of administration in e-prescriptions. The total prescribing errors in the OPD and emergency ward of Hospital A were found to be 44% and 60%, respectively. In hospital B, the OPD had 39% medication errors and the emergency department had 73.5% errors; this unexpected difference between the emergency ward and OPD of hospital B was mainly due to the inclusion of 69.4% omissions of route of administration in the prescriptions. The incidence of prescription overdose was approximately 7%–19% in the manual system and approximately 8% in semi and fully electronic system. The omission of information and incomplete information are contributors of prescribing errors in both manual and electronic prescriptions.

## Introduction

A medication can be a blessing if the healthcare provider prescribes, dispenses and administers drugs to patients correctly. Despite the best efforts, medication errors occur every day all over the world, which may be detrimental to patient well being. The National Coordinating Council for Medication Error Reporting and Prevention (NCC MERP) reported that approximately 0.1 million people die annually from medical errors that occur in hospitals and the resulting death toll/year due to medication errors is higher than that of work place injuries [1]. Beside loss of life, such errors can result in unwanted outcomes, loss of confidence in the healthcare system, increase in treatment cost and longer stay in hospitals [2]. In developed countries like the US, such errors are reported to be responsible for 7000 deaths per year [3].

Medication errors (MEs) may occur by both medical and paramedical personnel at various levels of patient care, hence multilevel monitoring is required. There are many reports of errors in medication committed by nurses [4]–[5]. Even in intensive care units (ICUs), where the medical and paramedical personnel are more skilled, the frequency of medication errors is reported to be 52.5% [6]. Many attempts are being made to reduce the occurrence of medication errors particularly by the use of information technology e.g. e-prescribing, which has significantly reduced the chances of such errors [7]–[9].

In Pakistan, some hospitals have adopted e-prescribing fully or partially to reduce medication errors but data on MEs is scant. In a study, the prevalence of transcription errors in a main public hospital in Pakistan was studied [10]. The present study was conducted to find the occurrence of medication errors in prescription writing (prescribing errors) from outpatient department and emergency ward of two public sector hospitals of Lahore, Pakistan. The secondary aim was to assess which age group and gender received most of the treatment. The findings of the present study may be useful in preventing prescribing errors and increasing confidence in health care system.

## Materials and Methods

### Ethics Statement

The study was approved by the Human Ethical Committee, University College of Pharmacy, University of the Punjab, Lahore. The written informed consent was not needed from the participants because de-identified administrative prescription records were used to get the data. Furthermore, patient’s information was anonymized and de-identified prior to data analysis.

### Sample and Data Collection

#### Study Design

The study was conducted to find and compare prescription errors in conventional manual prescriptions and e-prescriptions at two public hospitals designated as hospital-A and hospital-B. Hospital-A, has a capacity of 150 beds and deals with the general public. The average number of patients treated per day during 2012 was 1610 and 1209 in OPD and emergency departments respectively. In this hospital, all prescriptions are written manually. Hospital-B, deals government employees as well as general public. This hospital has 1096 beds and e-prescribing is used in emergency and partial e-prescribing in OPD where physicians write manually only the inscription part of prescription (body of prescription). The average number of patients treated per day during 2012 was 1504 and 1452 in OPD and emergency departments respectively.

A total of 510 prescriptions were studied for each OPD and Emergency of Hospital A and Hospital B in 34 days (not Sundays for OPDs) from June to July, 2012 and from September to October, 2012 respectively.

#### Collection of Prescriptions

Fifteen prescriptions were selected by simple random method, where all members of a population have equal chance of being selected, from a daily total collection of prescriptions (present in official record room) written by medical practitioners from OPD and emergency departments. In OPD, data were obtained from prescriptions written from various specialities. In emergency, data were collected from prescriptions of male, female and paediatrics wards of the emergency.

#### Data Recording

The data were recorded on data sheets. Prescriptions were evaluated for information for omission of patient’s information, legibility and mistakes. The recorded information included prescription number, patient’s name, patient’s address, age, gender, diagnosis, prescribing department and signature of prescriber. The inscription details were also recorded are given in Table 1.

**Table 1:** Inscription details evaluated.

Item	Evaluation				
Drug product selection	Correct	Incorrect			
Dose	Correct dose	Not mentioned	Illegible	Overdose	Underdose
Dosage form	Mentioned	Not mentioned	Illegible	Wrong	
Quantity	Mentioned	Not mentioned	Illegible		
Route of Administration	Mentioned	Not mentioned	Illegible	Wrong	
Concentration	Mentioned	Not mentioned	Illegible		
Rate of Administration	Mentioned	Not mentioned	Illegible	Wrong	
Legibility	Legible		Illegible		

#### Prescribing Error

Any omission/not-mentioned, wrong, incorrect or illegible information on prescription’s inscription part (part of the prescription containing the names and amount of ingredients) was considered as a prescribing error. Wrong gender was identified if the gender written on the prescription did not match with the name. Under dose, over dose, illegible or omitted were considered as dose errors.

Total prescribing errors included only errors in the inscription part of the prescription. These were equal to incorrect information on inscription+under dose+overdose+omission (on inscription part)+any illegibility in inscription part. Prescribing errors were found according to ASHP Guidelines on Preventing Medication Errors in Hospitals issued by American Society of Hospital Pharmacists [11].

#### Dose Error

Drug Dose Errors/Improper Dose Errors (over-dose/under-dose) were found by consulting British National Formulary BNF-61 [12] by the researcher (MKR). The amount over the normal range was considered overdose while below the range was underdose.

#### Statistical Analysis

The collected data were entered in Excel and then coded. The data were analysed by SPSS version 16 for Windows. Chi Square Test was employed to compare prescribing errors in OPD and emergency wards of the two hospitals. Prescribing errors were presented as percentage.

## Results and Discussion

### Comparative Errors in Out Patient Department

#### Errors in Patient Characteristics and Demographics

The patient characteristics such as name, age, gender, prescription No., address, diagnosis, prescribing department and prescriber’s signature are given in Table 2. About 90% prescriptions from OPDs of both the hospitals were found to be having description of gender. The prescriptions with prescriber’s signatures and prescription No. were about 95%. Diagnosis was noted on 31% and 46% prescriptions in Hospital A and Hospital B, respectively. Illegible names were 0% in Hospital B while 2% in Hospital A which appeared to be inattention, probably due to high number of patients and workload. Illegibility could be seen in name, gender, prescription No. and diagnosis particularly in Hospital A which used the manual prescription system. It has been shown that the presence of illegibility and incomplete information in prescriptions is a bad prescription writing practice and in this connection awareness should be increased among physicians [13].

**Table 2:** Patient characteristics in two hospitals.

Characteristic	Prescription Status	Frequency (%)			
		Outpatient		Emergency	
		Hosp A	Hosp B	Hosp A	Hosp B
	Mentioned	500 (98.0)	510 (100.0)	500 (98.0)	509 (99.8)
Name	Not Mentioned	0 (0)	0 (0)	0 (0)	1 (0.2)
	Illegible	10 (2.0)	0 (0)	10 (2.0)	0 (0)
	Male	226 (44.3)	252 (49.4)	189 (37.1)	305 (59.8)
	Female	232 (45.5)	257 (50.4)	273 (53.5)	188 (36.9)
Gender	Illegible	22 (4.3)	0 (0)	14 (2.7)	0(0)
	Not Mentioned	30 (5.9)	0(0)	34 (6.7)	0 (0)
	Wrong	0 (0)	1 (0.2)	0 (0)	17 (3.3)
	Mentioned	504 (98.8)	510 (100.0)	506 (99.2)	510 (100.0)
Prescription No.	Not Mentioned	2 (0.4)	0 (0)	1 (0.2)	0 (0)
	Illegible	4 (0.8)	0 (0)	3 (0.6)	0 (0)
	Mentioned	0 (0)	0 (0)	412 (80.8)	0 (0)
Address	Not Mentioned	510 (100.0)	510(100.0)	26 (5.1)	510 (100.0)
	Illegible	0 (0)	0 (0)	72 (14.1)	0 (0)
	Mentioned	158 (31.0)	236 (46.3)	342 (67.1)	0 (0)
Diagnosis	Not Mentioned	343 (67.2)	273 (53.5)	148 (29.0)	510 (100.0)
	Illegible	9 (1.8)	1 (0.2)	20 (3.9)	0 (0)
Prescriber’s Signature	Yes	498 (97.6)	486 (95.3)	359 (70.4)	510 (100.0)
	No	12(2.4)	24 (4.7)	151 (29.6)	0 (0)

#### Distribution of Age Groups and Prescribing Departments

**Figure 1:**
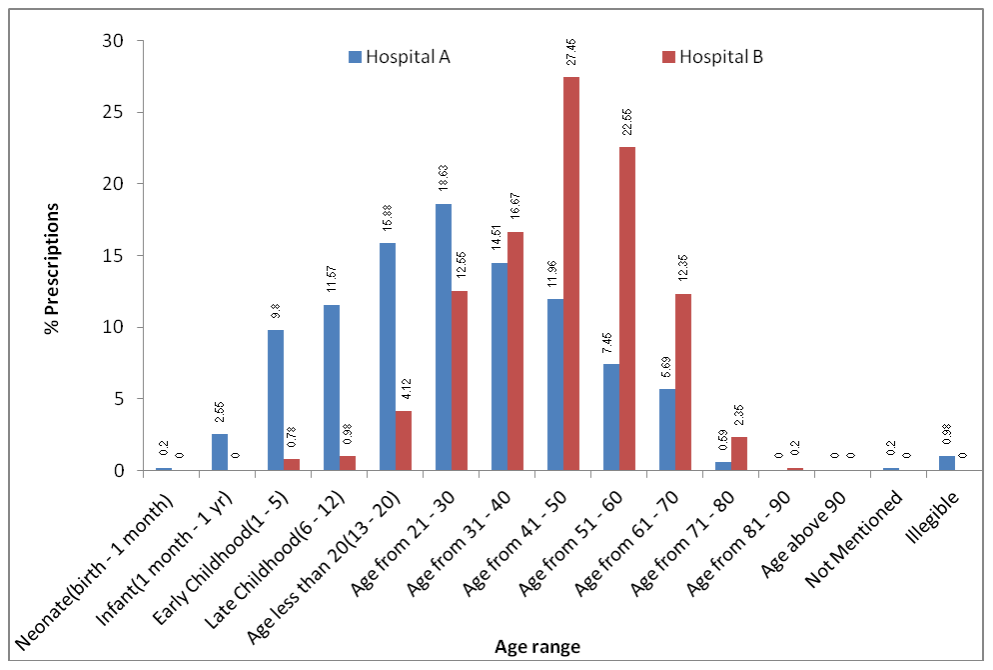
The patient age groups in outpatient departments of Hospital A and Hospital B.

**Figure 2:**
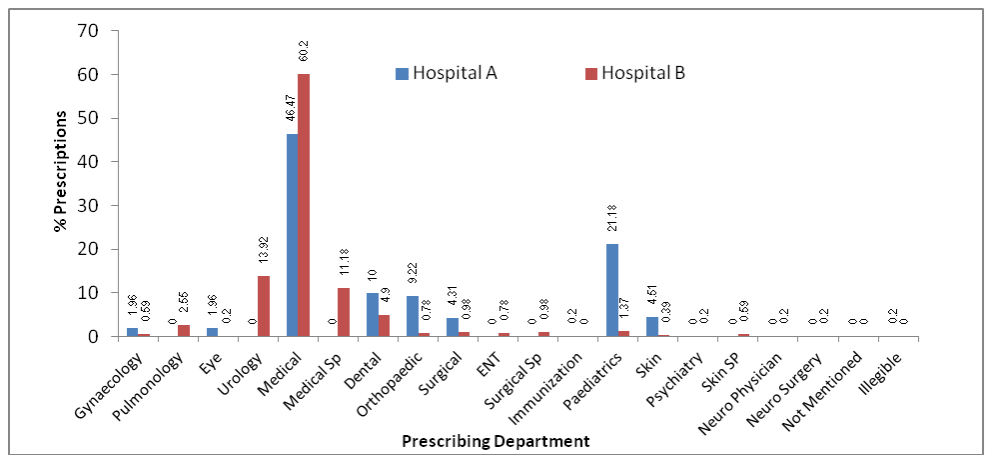
Prescribing departments in outpatient department of Hospital A and Hospital B.

The most age group visited Hospital A was 21–30 years (18.6%) and in Hospital B was 41–50 years with 27.5% visits (Figure 1). Hospital B received more patients in age range of 41–60 years than Hospital A because Hospital B mostly caters government employees. Hospital A received more patients in childhood to 30 years of age because it was situated in the centre of city. The most prescribing department in both hospitals was medical with 46.5% and 60.2% prescriptions for Hospital A and Hospital B respectively (See Figure 2).

#### Prescribing Errors

The inscription details of prescriptions in OPD of two hospitals are given in Table 3. The main error was omission of the required information and lesser number of prescriptions was with other errors. The prescriptions in Hospital A shown to have more number of errors for drug product selection (1.4%) and quantity (6.5%) compared to Hospital B; Hospital A has lesser errors for route of administration and dosage form. In a previous study, omission error and wrong dose error were shown to be 23.8%, 15.1% respectively [6].

**Table 3:** Prescription inscription details of Hospital A and Hospital B.

Item	Prescription Status	Frequency (%)			
		Outpatient		Emergency	
		Hosp A	Hosp B	Hosp A	Hosp B
Drug Product Selection	Correct	503 (98.6)	509 (99.8)	506 (99.2)	510 (100.0)
	Incorrect	7 (1.4)	1 (0.2)	4 (0.8)	0 (0)
	Mentioned	506 (99.2)	499 (97.8)	507 (99.4)	494 (96.9)
Dosage Form	Not Mentioned	2 (0.4)	11 (2.2)	2 (0.4)	16 (3.1)
	Wrong	2 (0.4)	0 (0)	0 (0)	0 (0)
	Illegible	0 (0)	0 (0)	1 (0.2)	0 (0)
	Mentioned	477 (93.5)	478 (93.7)	288 (56.5)	509 (99.8)
Quantity	Not Mentioned	33 (6.5)	32 (6.3)	219 (42.9)	1 (0.2)
	Illegible	0 (0)	0 (0)	3 (0.6)	0 (0)
	Mentioned	507 (99.4)	498 (97.6)	419 (82.2)	153 (30.0)
Route of Administration	Not Mentioned	3 (0.6)	10 (2.0)	68 (13.3)	354 (69.4)
	Illegible	0 (0)	0 (0)	20 (3.9)	0 (0)
	Wrong	0 (0)	2 (0.4)	3 (0.6)	3 (0.6)
	Mentioned	502 (98.4)	506 (99.2)	391 (76.7)	505 (99.0)
Concentration	Not Mentioned	8 (1.6)	4 (0.8)	115 (22.5)	5 (1.0)
	Illegible	0 (0)	0 (0)	4 (0.8)	0 (0)
	Mentioned	396 (77.6)	509 (99.8)	253 (49.6)	510 (100.0)
Rate of Administration	Not Mentioned	114 (22.4)	1 (0.2)	247 (48.4)	0 (0)
	Illegible	0 (0)	0 (0)	9 (1.8)	0 (0)
	Wrong	0 (0)	0 (0)	1 (0.2)	0 (0)
Legibility	Illegible	15 (2.9)	29 (5.7)	36 (7.1)	0 (0)
	Legible	495 (97.1)	481 (94.3)	474 (92.9)	510 (100.0)

Illegible prescriptions were 2.9% and 5.7% for Hospital A and Hospital B, respectively (Table 3). The prescriptions in Hospital B had average 5 medicines as compared to 3 in Hospital A. Furthermore, in Hospital B OPD inscription part of prescription was written manually. It has been found by Katzung (2006) that poor writing leads to errors of drug dose or drug administration timings [14].

### Comparative Errors in Emergency Departments

#### Errors in Patient Characteristics and Demographics

The patient characteristics of two emergencies are given in Table 2. The addresses of patients were mentioned on 81% and 0% prescriptions in emergency departments of Hospital A and Hospital B, respectively. The addresses of patients on the prescriptions from OPD of both hospitals were also not given. This is alarming because any potential prescribing error in prescriptions cannot be corrected.

In Hospital A, 37.1% prescriptions were for male and 53.5% for female. It was 59.8% for male and 36.9% for female in Hospital B. The rest of the information for gender was illegible or wrong. Hospital A has more female patients than that visited the Hospital B. Prescriptions with prescriber’s signature were noted to be 70.4% in Hospital A as compared to 100% in Hospital B. Prescription number mentioned was 99.2% and 100% for Hospital A and Hospital B, respectively.

Diagnosis was mentioned on 67% of the prescriptions in Hospital A while 0% of the in Hospital B (Table 2). In both OPDs, prescriptions with diagnosis mentioned were about 31% and 46% in Hospital A and Hospital B respectively. The general trend appears to be not writing diagnosis on the prescriptions. Diagnosis should be essential for safe treatment of patients and follow up. Illegible names were 2% in Hospital A and 0% in Hospital B, respectively. The prescriptions without names were 0.2% in Hospital B. The patient characteristics part of prescriptions was almost free of errors in electronic prescriptions than that of the manual prescriptions (Table 2).

#### Distribution of Age Groups

**Figure 3:**
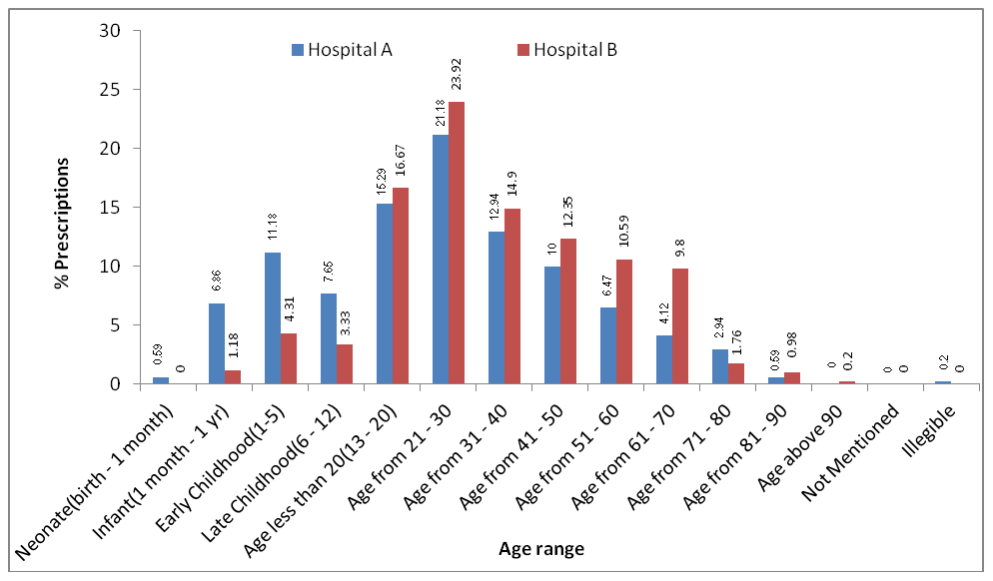
Hospital A and B Emergency Age Groups.

The age group of 21–30 years (21–24%) was found to be the highest the group which visited both hospitals for emergency treatments (Figure 3).

#### Prescribing Errors

Prescriptions inscription details for two emergencies are given in Table 3. Route of administration was not mentioned on 69.4% of prescriptions in emergency of Hospital B than on 13.3% of Hospital A emergency prescriptions. The illegible prescriptions were 7% and 0% for Hospital A and Hospital B, respectively. The use of computerized prescription system was the main reason for 100% prescriptions to be legible (Table 3). From the results it could be inferred that the use of electronic prescriptions can reduce the prescribing errors and hopefully increase the patient safety. Computerized Physician Order Entry (CPOE) has been shown to improve patient safety by reducing MEs and subsequent adverse drug events (ADEs). It was found that the largest reduction in errors was due to the use of CPOE in the areas of illegibility (97%), use of inappropriate abbreviations (94%) and missing information (85%) [15]. Due to this reason, the Spanish Society of Hospital Pharmacy has established a plan of action of introducing e-prescription system in 80% of hospitals up to 2020 [8]. However, the e-prescription is not without errors. In this connection Hayman *et al* proposed that the patient pictures should be used in the prescription system to reduce CPOE errors [16].

### Dose Error (Over-Dose/Under-Dose)

Dose in total 2040 prescriptions for both hospitals was checked from the point of view: within limits/no dose error, over dose, under dose, illegible and omitted dose (Table 4). These were found by matching the prescribed doses with that of the reported in BNF 61 [12].

**Table 4:** Dose at two hospitals (for each, n = 510).

Dose	Hospital A	Hospital B	Hospital A	Hospital B
	Outpatient Department (%)		Emergency (%)	
Within Limits	55.9	68.6	45.1	83.5
Under Dose	0.6	2.5	2.7	7.5
Over Dose	19.2	9.0	7.6	7.8
Not Mentioned	24.3	19.8	43.9	1.0
Illegible	0	0	0.6	0

There was a trend of over dose (7.6–19.2%) prescribing in both hospitals. At higher doses, undesired side effects appear and may become severe as the dose increases. Over dose, accidentally or due to prescribing error may cause from minor harm to permanent organ damage. In a testimony before a Senate subcommittee during 2008, according to Paulozzi ‘*more than 22,000 Americans lives are lost in 2005 due to over doses*’ [17]. Thus over dose prescribing should be avoided. In this connection, the role of the hospital pharmacists may be useful in controlling prescribing errors. Such a role of pharmacist has not been realized or utilized in Pakistan. In emergency of Hospital A, the prescriptions on which the dose was not mentioned counted as 43.9% as compared to 1% in emergency department of Hospital B, probably due to e-prescribing in this department. In a study, the most common types of error throughout the medication process were: lack of drug form, unordered drug, and omission of drug/dose [18]. A small percentage (0.6–7.5%) of under dose can be seen in both hospitals prescriptions, this may lead to incomplete treatment and the patients usually need extra visits to hospitals.

### Total Prescribing Errors

It was found that outpatient departments of Hospital A and Hospital B had 44% and 39% errors respectively while emergency of Hospital A and Hospital B had 60% and 73.5% total prescribing errors respectively (based on the data in Table 3). High values of total prescribing errors from 39 (outpatient department of Hospital B) to 73.5% (Emergency of Hospital B) were consistent with a previous studies in ICU by Agalu *et al.* [6]. They found that among the 398 drug prescriptions, there were 209 prescriptions containing at least one error and the frequency of prescribing errors was reported to be 52.5%.

There should be lesser number of errors in the emergency of the Hospital B which was using the computerized prescription system. Nevertheless, the emergency of Hospital B has total prescribing errors of 73.5% as compared to the 60% for the emergency department of Hospital A, opposite to what one can expect. This error was due to the inclusion of the prescriptions bearing no route of administration which was 69.4%. Total prescribing errors become lower (4.1% error) when the above error was deducted from the total prescription errors in emergency department of Hospital B. This indicates that prescribing errors in electronic prescriptions can be due to the omission of important prescription information. It has also been shown that writing incomplete information creates ambiguity and produces prescription errors [19].

Due to higher percent of prescribing errors in hospitals, a medication error reporting system should be established in hospitals of Pakistan as majority of the medication errors remain unnoticed. This system will be extremely beneficial, in general for patients of all age groups and, in particular for the most vulnerable age groups such as children and elderly patients. Reporting of these errors will help to prevent medication errors in future. Such systems exist at national and local levels in 16 countries where the medication errors are very less, if any [20].

### Statistical Analysis

The results of Chi-Square Test are given in Table 5. A p value ≤0.05 was taken as significant difference.

**Table 5:** Statistical analysis.

Patient Characteristics	OPDs of two hospitals		Emergencies of two hospitals	
	Probability	Significance	Probability	Significance
Name	0.00	Sig.	0.01	Sig.
Age	0.00	Sig.	0.00	Sig.
Gender	0.00	Sig.	0.00	Sig.
Prescription No.	0.01	Sig.	0.13	Non Sig.
Diagnosis	0.00	Sig.	0.00	Sig.
Prescribing Dept	0.00	Sig.		
Prescriber’s Sig.	0.04	Sig.	0.00	Sig.
**Inscription Details**	**OPDs of two hospitals**		**Emergencies of two hospitals**	
	**Probability**	**Significance**	**Probability**	**Significance**
Incorrect Product Selection	0.03	Sig.	0.08	Non Sig.
Dose	0.00	Sig.	0.00	Sig.
Dosage Form	0.03	Sig.	0.00	Sig.
Quantity	0.90	Non Sig.	0.00	Sig.
Route of Admn	0.05	Sig.	0.00	Sig.
Concentration	0.25	Non Sig.	0.00	Sig.
Rate of Admn	0.00	Sig.	0.00	Sig.
Illegible Prescriptions	0.03	Sig.	0.00	Sig.
**Total prescribing errors**				
Two OPDs	0.09	Non Sig.		
Two Emergencies	0.00	Sig.		

Note: OPD = Out Patient Department and Sig. = Significant.

The study has some limitations. Medication error (ME) is a very wide definition. ME can be an error in the process of prescribing, transcribing, dispensing and administration of drugs [18]. We have investigated only prescribing errors which deal with errors in prescription writing. However, the present study provides useful information about such errors in the prescriptions of public sector hospitals and may also be helpful in preventing prescribing errors.

## Conclusions

The occurrence of prescribing errors was found in both public sector hospitals. There is a trend of omitting address and diagnosis in almost all prescriptions in both of the hospitals. The incidence of overdose was found to in both the hospital. The medical departments of the hospitals are the major prescribers. The most age group receiving treatments in emergencies was of 21–30 years.
